# Supplementary material for: The genome sequence of the Purple-edged Copper, Lycaena hippothoe (Lepidoptera: Lycaenidae)
Source: Wellcome Open Res. 2025 Aug 22;10:458. [Version 1] doi: 10.12688/wellcomeopenres.24747.1 (PMC12547381; doi:10.12688/wellcomeopenres.24747.1)
Supplement: Peer review history

# Reviewer report: Reviewer response for version 1

**DOI:** 10.21956/wellcomeopenres.27264.r131672

**Published:** 2025-10-22

**Authors:** Megan Barkdull

**Affiliations:** 1 Cornell University, Ithaca, New York, USA; 2 Entomology, Natural History Museum of Los Angeles County (Ringgold ID: 5119), Los Angeles, California, USA

The authors present a genome assembly for the Lycaenid butterfly Lycaena hippothoe. 

 My most substantial criticism is that the authors have not indicate where the voucher specimen pictured in Fig. 1 has been deposited. It should be deposited in a recognized natural history collection to permit future study. 

 I indicated that the authors did not convey a rationale for creating the dataset; although they did not, in my view simply providing additional genomic resources to the research community is sufficient rationale. 

 It would be helpful if the authors could add collecting permit information or indicate that permits were not necessary. 

 The colors in Fig. 3 are extremely difficult to distinguish, and I urge the authors to adopt a different color scheme that allows the 31 Merian elements to be more clearly identified.

Are sufficient details of methods and materials provided to allow replication by others?

Yes

Is the rationale for creating the dataset(s) clearly described?

No

Are the datasets clearly presented in a useable and accessible format?

Yes

Are the protocols appropriate and is the work technically sound?

Yes

Reviewer Expertise:

Comparative genomics, entomology.

I confirm that I have read this submission and believe that I have an appropriate level of expertise to confirm that it is of an acceptable scientific standard, however I have significant reservations, as outlined above.

# Reviewer report: Reviewer response for version 1

**DOI:** 10.21956/wellcomeopenres.27264.r131668

**Published:** 2025-09-23

**Authors:** David Lohman

**Affiliations:** 1 The City College of New York Department of Biology (Ringgold ID: 171428), New York, USA; 2 Entomology Section, National Museum of Natural History, National Museum of the Philippines (Ringgold ID: 198024), Manila, Philippines; 3 Graduate Center, PhD Program in Biology, City University of New York (Ringgold ID: 2009), New York, USA

This concise genome note summarizes the biology of the widespread European copper butterfly *Lycaena hippothoe* before explaining the methods that resulted in a highly complete genome assembled at the chromosome level. Since the primary aim of this note is to describe and publicize the genome, the brevity of the introduction and lack of hypotheses is not a problem.  Nevertheless, I suggest the addition of a few sentences of additional text. Host ant associations of lycaenid larvae (or the absence of such associations) is nearly as ecologically important as data on host plant association. Therefore, I would suggest that the following text be added after the sentences on host plants and before the text on voltanism.

 "Unlike some of its close relatives, larvae of *L. hippothoe* have never been recorded in association with ants. In contrast, larvae of Palearctic *Lycaena dispar* facultatively associate with ants from two different subfamilies (Fiedler 2021). Larvae of *Tharsalea xanthoides, T. edita, T. heteronea,* and *T. rubidus *have also occasionally been recorded with ants (Ballmer & Pratt 1988; Fiedler 2021). These latter four species were previously placed in the genus *Lycaena *before the recent subfamily revision of Lycaeninae by Zhang et al. (2023)."

 By citing the Zhang et al. paper, you are also acknowledging the existence of this revision published in a somewhat obscure journal, and tacitly confirm that *hippothoe* is still in *Lycaena.*

 Aside from that addition of text, I would suggest the following rather minor changes

 - I would write "northern Spain", not "Northern Spain" because the area is not a formal place name. Later in the text "northern Iran" is used.

 -Add that the host plant family is Polygonaceae

 -Change "Larva overwinters" to "Larvae overwinter"

 -Change "The species forms one adult..." to "The species is univoltine with one adult..."

 -Remove "considered" in the phrase " *L. hippothoe* is considered of least concern"

 -After "..sequenced as part of Project Psyche" cite the pre-print and/or website

 -Figure 1 - Is it possible to show the wing uppersides, too?

 -Perhaps cite an appropriate reference to explain what Hi-C is.

 -In this phrase "enrichment" does not need to be capitalized: "...DNA remains bound to the Enrichment beads..."

 -Provide a citation explaining what "Chromosome painting" is.

 -The GC content seems slightly higher in the 2nd contig, which seems to be the W chromosome. This might merit some discussion or speculation. If this is common in lepidopteran W chromosomes, this should be mentioned and cited.

 -Fig. 6 - Dichotomizing the reads into Arthropod/non-Arthropod seems to be lost opportunity to look for *Wolbachia* and other endosymbiotic bacteria.

 -In the reference Trense et al 2022a, "lycaenid" should not be capitalized because it is not a proper noun; this is an adjective.

 Reference:

 3. Zhang, J., Q. Cong, J. Shen, L. Song, and N. V. Grishin. 2023. Butterfly classification and species discovery using genomics. The Taxonomic Report of the International Lepidoptera Survey 11:1-94. https://digitalcommons.unl.edu/taxrpt/88/

**https://doi.org/10.5281/zenodo.8400884**

Are sufficient details of methods and materials provided to allow replication by others?

Yes

Is the rationale for creating the dataset(s) clearly described?

Yes

Are the datasets clearly presented in a useable and accessible format?

Yes

Are the protocols appropriate and is the work technically sound?

Yes

Reviewer Expertise:

Ecology, evolution, systematics, and biogeography of butterflies

I confirm that I have read this submission and believe that I have an appropriate level of expertise to confirm that it is of an acceptable scientific standard.

## References

[1] : A survey of the last instar larvae of the Lycaenidae (Lepidoptera) of California. *The Journal of Research on the Lepidoptera* .1988;27(1) : 10.5962/p.332211 1-81 10.5962/p.332211

[2] : The ant associates of Lycaenidae butterfly caterpillars – revisited. *Nota Lepidopterologica* .2021;44: 10.3897/nl.44.68993 159-174 10.3897/nl.44.68993

# Reviewer report: Reviewer response for version 1

**DOI:** 10.21956/wellcomeopenres.27264.r133132

**Published:** 2025-09-22

**Authors:** Ozren Polašek

**Affiliations:** 1 University of Split, Split, Croatia

Dear authors, I recently reviewed another of your submissions, and I would suggest to do a similar protocol  in here as well. The molecular work does not seem to have any major limitations or problems, but I am again inclined to double check the taxonomic situation to be sure. The photo that you provided does not show sufficient detail for me to be convinced about the species, so please try to provide two more things. The first is to show the wing underside, if you still have it. The second is to consider using mtDNA/COI to validate the species against BOLD. That would reduce the chance of an error and strengthen the overall message.

Are sufficient details of methods and materials provided to allow replication by others?

Yes

Is the rationale for creating the dataset(s) clearly described?

Yes

Are the datasets clearly presented in a useable and accessible format?

Yes

Are the protocols appropriate and is the work technically sound?

Yes

Reviewer Expertise:

Taxonomy, genetics

I confirm that I have read this submission and believe that I have an appropriate level of expertise to confirm that it is of an acceptable scientific standard, however I have significant reservations, as outlined above.
